# Contrast-Enhanced Computed Tomography–Based Radiogenomics Analysis for Predicting Prognosis in Gastric Cancer

**DOI:** 10.3389/fonc.2022.882786

**Published:** 2022-06-22

**Authors:** Han Liu, Yiyun Wang, Yingqiao Liu, Dingyi Lin, Cangui Zhang, Yuyun Zhao, Li Chen, Yi Li, Jianyu Yuan, Zhao Chen, Jiang Yu, Wentao Kong, Tao Chen

**Affiliations:** ^1^ Department of Ultrasound, The Affiliated Drum Tower Hospital of Nanjing University Medical School, Nanjing, China; ^2^ Department of General Surgery, Nanfang Hospital, Southern Medical University, Guangdong Provincial Engineering Technology Research Center of Minimally Invasive Surgery, Guangzhou, China; ^3^ School of Biomedical Engineering, Southern Medical University, Guangzhou, China; ^4^ Department of Radiology, Southern Medical University, Guangzhou, China

**Keywords:** radiogenomic, gastric cancer, nomogram, radiomic, prognosis

## Abstract

**Objective:**

The aim of this study is to identify prognostic imaging biomarkers and create a radiogenomics nomogram to predict overall survival (OS) in gastric cancer (GC).

**Material:**

RNA sequencing data from 407 patients with GC and contrast-enhanced computed tomography (CECT) imaging data from 46 patients obtained from The Cancer Genome Atlas (TCGA) and The Cancer Imaging Archive (TCIA) were utilized to identify radiogenomics biomarkers. A total of 392 patients with CECT images from the Nanfang Hospital database were obtained to create and validate a radiogenomics nomogram based on the biomarkers.

**Methods:**

The prognostic imaging features that correlated with the prognostic gene modules (selected by weighted gene coexpression network analysis) were identified as imaging biomarkers. A nomogram that integrated the radiomics score and clinicopathological factors was created and validated in the Nanfang Hospital database. Nomogram discrimination, calibration, and clinical usefulness were evaluated.

**Results:**

Three prognostic imaging biomarkers were identified and had a strong correlation with four prognostic gene modules (P < 0.05, FDR < 0.05). The radiogenomics nomogram (AUC = 0.838) resulted in better performance of the survival prediction than that of the TNM staging system (AUC = 0.765, P = 0.011; Delong et al.). In addition, the radiogenomics nomogram exhibited good discrimination, calibration, and clinical usefulness in both the training and validation cohorts.

**Conclusions:**

The novel prognostic radiogenomics nomogram that was constructed achieved excellent correlation with prognosis in both the training and validation cohort of Nanfang Hospital patients with GC. It is anticipated that this work may assist in clinical preferential treatment decisions and promote the process of precision theranostics in the future.

## Introduction

Although the incidence of gastric cancer (GC) has decreased over the last 3 decades, it remains the second leading cause of cancer-related death worldwide and the most prevalent cancer in eastern Asia, whose burden is still substantial (1). Patients experience a high rate of tumor recurrence (20%–40%) although following treatment with traditional standard therapies—surgical resection plus adjuvant chemotherapy or radiochemotherapy (2, 3). The American Joint Committee on Cancer (AJCC) tumor-node-metastasis (TNM) staging system is currently the most commonly used tumor staging system worldwide and is considered to be the most valuable method for assessing the prognosis of malignant tumors (4). However, large variations in clinical outcomes have been shown in patients with the same stage and similar treatment regimens (5, 6). These findings suggest that the present GC staging system provides inadequate prognostic information and does not reflect the biological heterogeneity of GC (7). Therefore, identifying additional effective prognostic model that owns incremental value to the TNM staging system is necessary to achieve individualized medical treatments.

A radiogenomic analysis aimed at investigating molecular biomarkers can identify imaging traits that correspond to different molecular phenotypes with clinical and biologic relevance. It represents the evolution of the radiology–pathology correlation from the tissue level to the subcellular level and can compensate for the deficiency that the biological interpretations of imaging traits are lacking. Radiogenomics analysis can hold the promise to meet the clinical demand noninvasively and comprehensively, compared to biopsy with limited specimens, because the extensive imaging features for each patient can comprehensively describe the tumor phenotype characteristics. This non-invasive method can be performed repeatedly and is therefore eminently suitable for treatment follow-up.

Recent advances in the radiogenomics of other cancers, such as hepatocellular carcinoma (8), non–small cell lung cancer (9), glioblastoma multiforme (10), breast cancer (11), and liver cancer (12), have confirmed the potential synergy of integrating imaging and genomic data. However, thus far, few studies have investigated the radiogenomics of GC.

A nomogram is based on multivariate regression analysis and includes important influencing factors related to tumor prognosis. The nomogram has become the focus of interest in cancer research in recent years and is considered a useful tool for quantifying risk (13, 14). Therefore, this study aimed to develop a predictive model of the overall survival (OS) nomogram based on the radiogenomic features combined with the clinicopathological characteristics to predict prognosis and validate its incremental prognostic value to the traditional TNM staging system.

## Materials and Methods

### Data Source

We studied 407 patients with GC whose data (including RNA sequencing data and clinical information) was obtained from The Cancer Genome Atlas (TCGA) (15)—a landmark cancer genomics program that contains a large amount of genomic and clinical datasets. RNA sequencing data were normalized using the fragments per kilobase transcriptome per million reads method. Among the 407 patients with GC, 46 patients with contrast-enhanced computed tomography (CECT) imaging data were obtained from The Cancer Imaging Archive (TCIA) (16)—a service that hosts a large archive of medical images of cancer accessible for public download. Most patients underwent imaging on a multi-detector row CT scanners: 4-, 16-, or 64-slice CT scanners with slice thickness of 2.5~5 mm at 120 kVp and 200~500 mA.

In addition, because the number of the patients in TCGA database was limited (there were only 46 patients who owned imaging data), we obtained external 424 patients with GC with imaging data in Nanfang Hospital to develop a prognostic nomogram model. Thirty-two patients were excluded for the invisible lesion or insufficient image quality, and then 392 patients with GC were included in further research. All of these patients underwent partial or total radical gastrectomy and did not receive other therapy preoperatively. All of the contrast-enhanced abdominal CT images were acquired within 30 days before surgical resection; the detailed inclusion and exclusion criteria were described in our previous study (17). Patient characteristics are detailed in **Table 1**.

**Table 1:** Clinicopathologic characteristics of patients.

Variables	TCGA Cohort (n = 45)	Nanfang Cohort(Training) (n = 196)	Nanfang Cohort (Validation) (n = 196)	P-value
OS (days)	780.64 ± 472.38	894.49 ± 698.54	916.22 ± 711.10	0.76
Age (years)	65.00 ± 9.17	55.43 ± 10.87	54.59 ± 11.00	0.45
Sex (male)	39 (84.8%)	136 (69.4%)	139 (70.9%)	0.74
Pathological lymph node positive detection rate	0.25 ± 0.27	0.22 ± 0.27	0.22 ± 0.28	1.00
T stage				0.28
T1	0	34 (17.3%)	29 (14.8%)	
T2	1 (2.2%)	23 (11.7%)	17 (8.7%)	
T3	25 (55.6%)	24 (12.2%)	17 (8.7%)	
T4	19 (42.2%)	115 (58.7%)	133 (67.9%)	
N stage				0.81
N0	10 (22.2%)	63 (32.1%)	62 (31.6%)	
N1	9 (20.0%)	37 (18.9%)	35 (17.9%)	
N2	12 (26.7%)	38 (19.4%)	34 (17.3%)	
N3	14 (31.1%)	58 (29.6%)	64 (32.7%)	
Unknown	0	0	1 (5%)	
M stage				0.31
M0	43 (95.6%)	190 (96.9%)	193 (98.5%)	
M1	2 (4.4%)	6 (3.1%)	3 (1.5%)	
TNM Stage^a^				0.70
I	1 (2.2%)	41 (20.9%)	37 (18.9%)	
II	8 (17.8%)	35 (17.9%)	33 (16.8%)	
III	31 (68.9%)	96 (49.0%)	107 (54.6%)	
IV	5 (11.1%)	24 (12.2%)	19 (9.7%)	
Lauren’s classification				0.85
Intestinal	40 (88.9%)	35 (17.9%)	36 (18.4%)	
Diffuse	0	51 (26.0%)	55 (28.1%)	
Mixed	5 (11.1%)	9 (4.6%)	6 (3.1%)	
unknown	0	101 (51.5%)	99 (50.5%)	

Data are expressed as mean ± standard deviation or number (%); there are no significant differences between the training and validation cohorts in Nanfang Hospital database (P > 0.05).

a According to the 8th edition of the American Joint Committee on Cancer classification.

### Imaging Feature Extraction

To avoid artificial and subjective deviation, two professional abdominal radiologists cooperated to delineate each tumor using the Medical Imaging Interaction Toolkit (MITK) (version 3.6.0 Apr. 1, 2017) without referring to any clinical information. Four samples are shown in **Supplementary Figure S1**. Forty-four imaging features were extracted, including four first-order statistics and 40 texture features, from the delineated tumor outlines for each person using MATLAB automatically. Four different matrices, including the gray-level cooccurrence matrix (GLCM) (18), the neighborhood gray-tone difference matrix (NGTDM) (19), the gray-level size zone matrix (GLSZM) (20), and the gray-level run-length matrix (GLRLM) (21), were utilized to evaluate the 40 texture features of the regions of interest (ROIs). A detailed description of the four matrices and the features is shown in **Supplementary Table S1**.

Intra- and interclass correlation coefficients (ICCs) were used to assess the intra- and interobserver reproducibility of radiomics feature extraction. Thirty images were chosen randomly for ROI segmentation and feature extraction. The ROI segmentation was performed by two radiologists with, respectively, 12 (reader 1) and 8 years (reader 2) of experience in gastric CT interpretation. Reader 1 then repeated the same procedure 1 week later. An ICC greater than 0.75 indicates good agreement of the feature extraction. To ensure the independence of imaging features, we removed the features with high correlation. We calculated the mean absolute correlation using the “findCorrelation” function in the caret package (version 6.0-84) in R software. When the pairwise correlation was greater than 0.8, the feature was identified as the one with redundancy and will be eliminated.

### Gene Module Clustering

Compared with traditional analysis methods, which focus on a single gene or a few genes, we used weighted gene coexpression network analysis (WGCNA) (22), a systematic biological method to describe the pattern of gene association between different samples and obtain highly synergistic gene sets. The distinct advantage is that WGCNA converts an enormous amount of highly correlated genes into modules defined as clusters of densely interconnected genes to eliminate the problem of multiple hypothesis testing corrections.

WGCNA methodology can be used to construct a gene coexpression network, identify modules using hierarchical clustering and the dynamic tree cut method, study module relationships using eigengene networks, and obtain hub genes—the key regulators of each module. Because of the early advantages, the application of the WGCNA method has therefore been extended to variable types of high-throughput datasets, such as proteomic and metabolomic datasets, in recent years (23–25). To ensure the reliability of the prognostic gene modules, all the 407 patients with gene expression data were included.

### Survival Analysis and Functional Enrichment of Gene Modules

The survival analysis of a time-to-event outcome is a widely applied method to predict survival from a set of patient predictors or covariates. Univariate Cox proportional hazards regression (26) was used to identify the gene modules with significant prognostic ability in OS, defined as the time from pathology diagnosis to the date of death or the last follow-up. For the module eigengene of gene modules that were continuous, the contributions to OS were investigated by Kaplan–Meier survival analysis (log-rank test). 

We annotated the biological functions of the survival-related modules of GC using Gene Ontology (GO) enrichment analysis and Kyoto Encyclopedia of Genes and Genomes (KEGG) pathway analysis, allowing the identification of common biologic pathways from a public database. GO and KEGG analyses were performed using the clusterProfiler package (27, 28) in R. In addition, we calculated the false discovery rate (FDR) using the Benjamini–Hochberg method (29) to correct the multiple hypothesis testing of the survival analysis.

### Development and Ability Evaluation of Imaging Features

We utilized Spearman rank correlations to build a radiogenomic map combining selected imaging features and prognostic gene modules. The FDR was used again to correct for multiple testing. The imaging features that showed high correlation with the prognostic gene modules (P < 0.05, FDR < 0.05) were further analyzed by the univariate Cox proportional hazards model to assess the prognostic significance. Afterward, a bootstrap approach (30) was used to assess whether the prognostic signature had significant power compared with random chance (C-index = 0.5). For 100 times, we calculated the C-index of the imaging signature based on 35 randomly selected samples with correct outcome data and on 35 randomly chosen samples with random outcome data. The Wilcoxon test was applied to assess the differences between the two distributions (imaging signature and random chances).The prognostic signature with significant power (P < 0.05) was identified as the imaging biomarker.

### Generation of the Radiomics Score

On the basis of the prognostic imaging biomarkers identified from the TCGA database, the radiomics score (RIS) was constructed using a multivariate Cox proportional hazards regression model. The equation of RIS is detailed in the **Supplementary Material**. Because the median RIS is convenient for clinical application, it was used as a cutoff value to divide the patients into low‐risk and high‐risk groups. The log-rank test (Mantel–Cox test) was used to compare the difference in the survival status between two groups. We calculated RIS both in the TCGA database and Nanfang Hospital database, and log-rank test was used to identify their prognostic value. To discuss the prognostic significance of the imaging biomarkers and RIS, we tried to explain it from the perspective of immunity. We utilized the CIBERSORT algorithm (an algorithm that calculates the ratios of infiltrated immune cells) to investigate the immune infiltration level in patients with GC.

### Construction and Validation of the Nomogram

To compensate for the limitation of the number of patients in TCGA database, we utilized the database from Nanfang Hospital to construct a prognostic nomogram model for its large samples. We divided patients from Nanfang Hospital into training and validation cohorts in a ratio of 1:1 using the stratified randomization method. The training cohort was used to construct the nomogram that integrated both the RIS and significant clinicopathological characteristics, and the validation cohort was used to identify the predictive accuracy of the nomogram. The nomogram was constructed using “rms” package (31) of R software. With the application of the bootstrap method (1,000 replicates), a calibration curve was used to visualize the deviation of predicted probabilities from what actually happened. Moreover, decision curve analysis (32) was employed to quantify the clinical utility and compare the nomogram that we created with the TNM staging system. Finally, the receiver operating characteristic curve (ROC) analysis (33) was performed to measure the predictive performance of the nomogram score and the TNM stage. The Delong test was used to assess the differences between the two models.

### Statistical Analysis

Only two-sided P-value < 0.05 was considered to have necessary to statistic. The statistical analysis was implemented on R software (version 4.0.4). Several packages including ggplot2, rms, survival, survivalROC, survminer, nomogramFormula, and rmda were used to perform other statistical calculations and graphical work.

## Results

### Imaging Feature Extraction

We extracted 44 imaging features from each person, and 16 imaging features were removed because their pairwise correlation was greater than 0.8. The correlation matrix is shown in **Supplementary Figure S2**. The intraobserver ICCs ranged from 0.812 to 0.978, and the interobserver ICCs ranged from 0.786 to 0.912, indicating a favorable intra- and interobserver feature extraction reproducibility.

### Gene Module Clustering and Function Annotation

**Figure 1:**
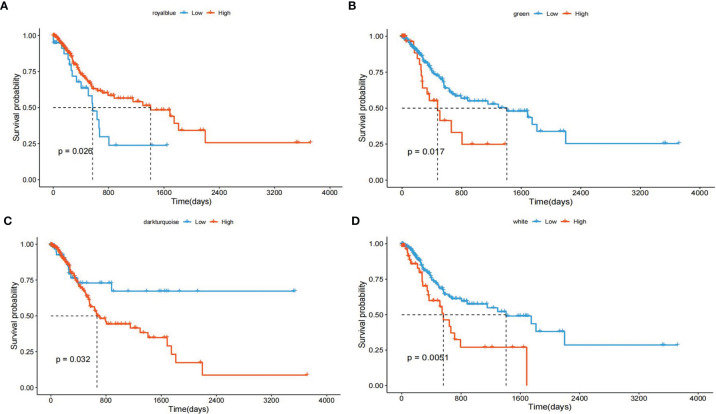
Kaplan–Meier plot with the univariate survival analysis of four gene modules. **(A)** The Kaplan–Meier plot shows that patients with a higher “MEdarkturquoise” value have shorter OS (blue lines), and patients with a lower “MEdarkturquoise” value have longer OS (red lines). **(B)** The Kaplan–Meier plot shows that patients with a higher “MEgreen” value have shorter OS (blue lines), and patients with a lower “MEgreen” value have longer OS (red lines). **(C)** The Kaplan–Meier plot shows that patients with a higher “MEroyalblue” value have shorter OS (blue lines), and patients with a lower “MEroyalblue” value have longer OS (red lines). **(D)** The Kaplan–Meier plot shows that patients with a higher “MEwhite” value have longer OS (blue lines), and patients with a lower “MEwhite” value have shorter OS (red lines).

We grouped transcripts with correlated expression levels into gene coexpression modules using the WGCNA approach. The cluster dendrogram and gene counts for each module are shown in **Supplementary Figure S3A**. Analysis of network topology for various soft-thresholding powers is shown in **Figures S3B, C**. The soft thresholding power β was set at 9 because the scale independence reached 0.8 (**Figure S3B**) and had a relatively high-average connectivity (**Figure S3C**). At last, we identified 33 gene modules among 58,428 genes. Across the 33 modules, four prognostic modules (MEgreen, MEwhite, MEdarkturquoise, and MEroyalblue) were identified using univariate Cox proportional hazards regression (**Table 2**), and the Kaplan–Meier plots of the four prognostic modules are shown in **Figure 1**. Afterward, four modules were tested for the enrichment of specific biological functions in GO terms and KEGG pathways. The results are shown in **Supplementary Table S2**. For example, the gene module MEdarkturquoise, enriched in protein kinase activator activity, was strongly correlated with a poor prognosis (P < 0.05). The gene module MEwhite, enriched in NADH dehydrogenase activity, was strongly correlated with positive survival.

**Table 2:** Gene modules with significant prognosis in OS.

Gene Module	Cox p	Hazard Ratio	CI (Lower–Upper)
MEdarkturquoise	0.0013	29.25	3.74–229.10
MEgreen	0.0058	13.96	2.14–90.94
MEroyalblue	0.0415	148.10	1.21–18,094.00
MEwhite	0.0331	1.60e−05	6.50e−10–0.41

CI, confidence interval.

### Radiogenomic Correlation

**Figure 2:**
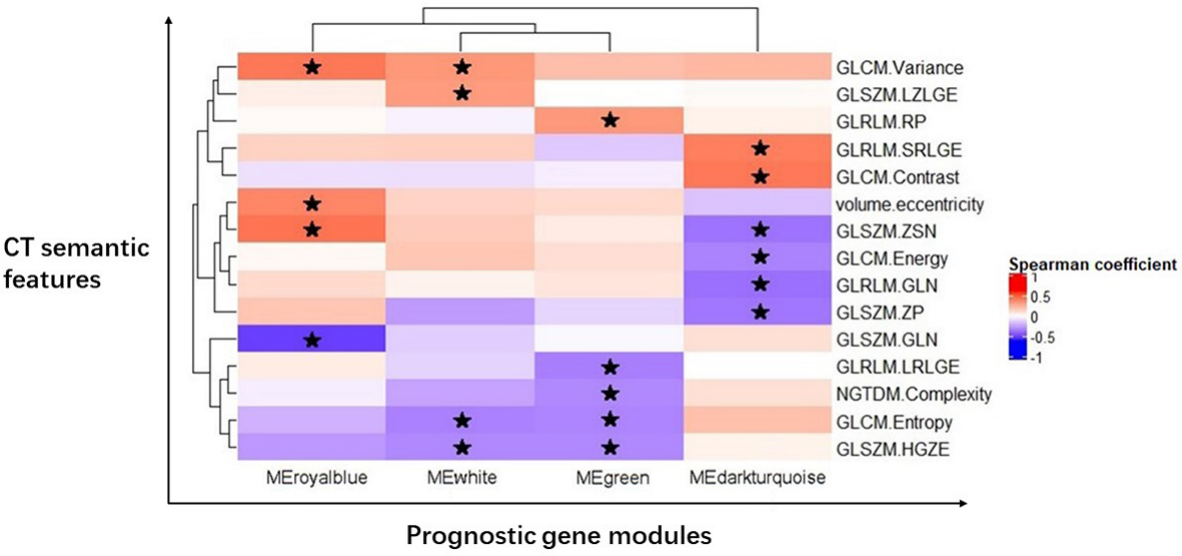
Radiogenomic map revealing 19 statistically significant associations between 15 CT semantic features and four prognostic gene modules in GC. Significant pairwise correlations (P < 0.05) are indicated with star symbols. The corresponding correlation coefficients are displayed in a heatmap; the red color indicates positive correlations, and the blue color indicates negative correlations.

We created the radiogenomic map that combined the remaining imaging features and four prognostic gene modules using Spearman rank correlations (**Figure 2**).

One first-order statistic (volume.eccentricity) and 14 texture features (GLCM.Energy, GLCM.Contrast, GLCM.Entropy, GLCM.Variance, GLRLM.GLN, GLRLM.RP, GLRLM.SRLGE, GLRLM.LRLGE, GLSZM.GLN, GLSZM.ZSN, GLSZM.ZP, GLSZM.HGZE, GLSZM.LZLGE, and NGTDM.Complexity) were identified to have a strong correlation with the gene modules (P < 0.05, FDR < 0.05). There were 19 pairs of significant correlations among the features and gene modules. For example, the gene module MEdarkturquoise was negatively correlated with GLCM.Energy (P = 0.0379), GLRLM.GLN (P = 0.0161), GLSZM.ZSN (P = 0.0181), and GLSZM.ZP (P=0.0224) and positively correlated with GLCM.Contrast (P = 0.0079) and GLRLM.SRLGE (P = 0.0115).

### Identification of Imaging Biomarkers and Radiomics Score

**Figure 3:**
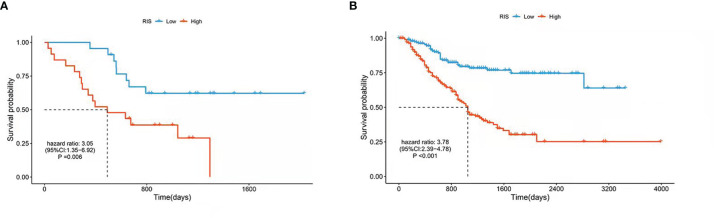
Kaplan–Meier estimates of the patients’ survival using the radiomics score. The Kaplan–Meier plots were used to visualize the patients’ survival probabilities for the low-RIS versus high-RIS group of patients based on the median radiomics score. **(A)** Kaplan–Meier curves for TCGA database patients (N = 45). **(B)** Kaplan–Meier curves for patients from Nanfang Hospital database (N = 392). The differences between the two curves were determined by the two-side log-rank test.

Univariate Cox proportional hazards regression was employed to assess the survival relationship of the 15 imaging features with survival outcomes. Three texture features, namely, NGTDM.Complexity, GLRLM.LRLGE, and GLSZM.ZP (shown in **Table 3**), were significantly correlated with OS. By using the bootstrap method, the Wilcoxon test showed significant differences between the imaging features and random chances. Thus, the three imaging features were identified as the imaging biomarkers of GC. In the TCGA database, the RIS was constructed using a multivariate Cox proportional hazards regression model based on three imaging features and their corresponding coefficients (**Table S3**). Then, patients were divided into a low-RIS group (n = 22) and a high-RIS group (n = 23) according to the median RIS. Compared with the low-RIS group, high-RIS group had a significantly lower OS rate [Hazard Ratio (HR) = 3.05, 95% confidence interval (CI): 1.35–6.92] (**Figure 3A**). To verify the ability of the RIS in predicting the OS of patients with GC, we further validated our findings in the database from Nanfang hospital, which yielded the similar results as above (HR = 3.41,95% CI: 2.41–4.82) (**Figure 3B**). Furthermore, multivariate Cox regression analysis was performed to determine whether the RIS was an independent prognostic factor for patients’ OS in both databases. In TCGA database, the P-value of RIS was 0.003 (HR = 1.99, 95% CI: 1.27–3.12), and in the database from Nanfang hospital, the P-value of RIS was less than 0.001(HR = 2.04, 95% CI: 1.52–2.75). Thus, RIS could be identified as an independent prognostic factor. Correlation between the imaging biomarkers/RIS and immune infiltration level is shown in the **Supplementary Material**.

**Table 3:** Imaging features with significant prognostic value for OS.

Imaging Features	Cox p	Hazard Ratio	CI (Lower–Upper)	FDR	C-index	Wilcoxon Test p
NGTDM.Complexity	0.0055	0.87	0.79–0.96	0.0155	0.7040	0.0278
GLRLM.LRLGE	0.0333	25451.93	2.23–2.91e+08	0.0333	0.5330	0.0392
GLSZM.ZP	0.0103	3.497e+20	71659.00–1.707e+36	0.0155	0.6190	0.0150

CI, confidence interval; FDR, false discovery rate.

### Building a Predictive Radiomics Nomogram for OS Prediction in GC

**Figure 4:**
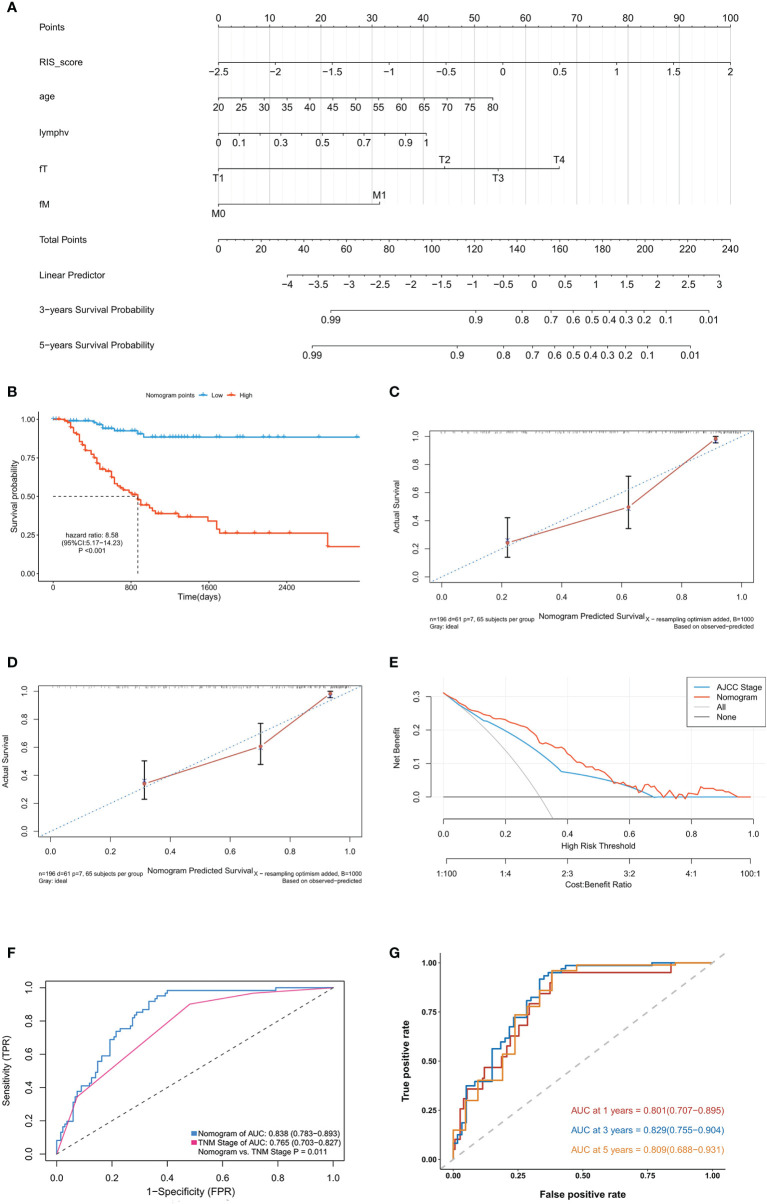
Development and validation of the raidiomics nomogram. **(A)** Nomogram constructed in conjunction with the radiomics score and clinical characterization that predict the 3- and 5-year overall survival of patients with gastric cancer. **(B)** Kaplan–Meier curves for patients with high and low nomogram score in the training cohort. **(C, D)** Plots depict the calibration of radiomics nomograms in terms of agreement between predicted and observed 3-year **(C)** and 5-year **(D)** outcomes. **(E)** Decision curves of the nomogram model and TNM stage for the survival predictions of patients with GC. **(F)** The ROC comparation between the nomogram model and the TNM stage. **(G)** Time‐dependent ROC analysis of the nomogram model for OS prediction in the training cohort. The area under the ROC curve was 0.803, 0.838, and 0.811 for the nomogram score at 1, 3, and 5 years, respectively.

In the training cohort of Nanfang Hospital database, the multivariate Cox analysis was employed to identify the independent prognostic factor in the RIS and clinicopathological characteristics. RIS, tumor metastasis, pathological lymph node positive detection rate, primary tumor, and age were significantly associated with OS (**Table 4**), and they were integrated to construct a nomogram (**Figure 4A**). After adding the sum of the points of the five variables, we can obtain the patients’ OS probabilities. Patients were divided into high– and low–nomogram point groups based on the median point. The Kaplan–Meier curve showed that the OS of the high–nomogram point group was significantly poorer [ hazarad ratio (HR) = 8.58, 95% CI: 5.17–14.23, P < 0.001] (**Figure 4B**). The calibration curve of our nomogram is shown in **Figures 4C, D**, which demonstrated the accurate predictive ability of the nomogram. Moreover, the nomogram model showed a better net benefit and broader threshold probability than the AJCC TNM staging system in the decision curves (**Figure 4E**), and if the threshold probability is between 0 and 0.53, then using the radiogenomics nomogram to predict OS adds more benefit than AJCC stage. ROC analysis revealed that the nomogram model (AUC = 0.838) had a better prognostic value compared to the TNM stage (AUC = 0.765) (**Figure 4F**). Moreover, the AUC value of 1-, 3-, and 5-year survival were 0.801 (95% CI: 0.707–0.895), 0.829 (95% CI: 0.755–0.904), and 0.809 (95% CI: 0.688–0.931), respectively (**Figure 4G**). It indicated that the nomogram model was a powerful tool for predicting patients’ survival.

**Table 4:** Multivariable Cox regression analysis of clinical pathological parameters in training cohort and the whole cohort.

	Trian Cohort		Whole Cohort	
Item	HR (95% CI)	P-value	HR (95% CI)	P-value
**RIS_score****^a^**	2.16 (1.40–3.32)	< 0.001	2.04 (1.52–2.75)	< 0.001
**Age****^a^**	1.03 (1.00–1.06)	0.02	1.02 (1.00–1.04)	0.013
**Lymphv****^a^**	4.07 (1.65–10.04)	0.002	7.34 (4.00–13.43)	< 0.001
**fT**				
T1	1.00 (Reference)	1.00	1.00	1.00
T2	4.60 (0.51–41.85)	0.18	1.87 (0.31–11.21)	0.49
T3	6.61 (0.68–64.28)	0.10	7.80 (1.61–37.74)	0.01
T4	9.98 (1.35–73.68)	0.024	6.34 (1.5–26.01)	0.01
**fM**				
M0	1.00	1.00	1.00	0
M1	2.96 (1.12–7.87)	0.03	2.06 (0.94–4.51)	0.07

a Continuous variable.

HR, hazard ratio; CI, confidence interval.

### Validation of the Radiomics Nomogram in the Validation Cohort

To further identify the predictive ability of the nomogram, we performed similar analysis in the validation cohort. The nomogram score for each patient was calculated in the same way (detailed in **Table S4**). Consisted with the findings mentioned above, Kaplan–Meier analysis showed that the patients in high–nomogram point group had a poor OS (HR = 4.93, 95% CI: 3.05–7.95, P < 0.001) (**Figure S4A**). Moreover, ROC analysis demonstrated that the nomogram (AUC = 0.791) model improved prognostic value in the validation compared with the TNM stage (AUC = 0.686) (**Figure S4B**). In addition, the time-dependent ROC showed that the nomgram model had a good accuracy with 0.843 (95% CI: 0.772–0.913) in 1 year, 0.772 (95% CI: 0.686–0.857) in 3 years and 0.767 (95% CI: 0.664–0.869) in 5 years (**Figure S4C**).

## Discussion

In this study, we integrated quantitative CT imaging features with RNA sequencing data to create a radiogenomic map and identified radiogenomic biomarkers of GC. The results revealed three prognostic imaging biomarkers and 19 significant pairwise associations between the imaging features and prognostic gene modules. Then, a RIS based on the three biomarkers was developed and proved to be independently associated with OS. A nomogram integrating both the RIS and clinicopathological characteristics performed better than the traditional TNM staging system, which demonstrated well the incremental value of the radiogenomic biomarker for individualized OS estimation.

The advantages of our radiogenomics analysis are as follows. First, radiogenomics analysis can hold the promise to meet the clinical demand noninvasively and comprehensively. Although the molecular biomarkers gained from preoperative biopsy have clinical benefit for prognosis of GC, the invasive approach may increase the potential risk to the patients. Moreover, compared with biopsy with limited specimens, we extracted extensive imaging features for each patient to comprehensively describe the tumor phenotype characteristics. In addition, there are barriers of high cost and technical problems when implementing invasive biopsy (34). Second, as for traditional radiomics research studies, the imaging features lack biological interpretations. Radiogenomics, combining the imaging features and molecular signatures, can compensate for this deficiency. In our study, prognostic value for clinical imaging with relevant molecular biology information is provided. Third, in contrast to traditional gene analysis methods, which focus on a single gene or a few genes and fail to reflect the association between genes, we employed WGCNA to divide 58,428 genes of each GC sample into 33 modules according to the correlation between genes. Therefore, complicated combinations and multiple testing problems can be avoided. In addition, we studied molecular prognostic significance by measuring OS predictors from public cohorts, which showed the prognostic associations of each gene module in GC. The three imaging biomarkers and correlations between the prognostic gene modules and imaging features were discussed in the **Supplementary Material**.

In the nomogram, RIS, tumor metastasis, lymph node positive detection rate, primary tumor, and age were kept after multivariate Cox analysis. The AUC of this radiogenomic nomogram is 0.838 in the training cohort and 0.791 in the validation cohort, which is higher than the previously published genomic nomogram (AUC = 0.744) by Chen et al. (35) and radiomic nomogram (AUC = 0.771) by Wang et al. (36). In addition, combining radiogenomic, clinical, and pathological information together produced this nomogram with better performance than using TNM staging information alone (**Figure 4F**). Thus, this comprehensive and individualized risk score calculation method could be used as stratification criteria in randomized studies and clinical trials.

Our study still had some limitations. First, the sample size of the CT images corresponding to gene expression profiles in TCGA database was not large enough, which may limit the reliability (8). Therefore, further studies with larger sample sizes are needed. However, to some extent, external validation from Nanfang Hospital was used to improve the dependability in this study. Second, the image datasets downloaded from the TCIA were extremely heterogeneous in terms of scanner modalities, manufacturers, and acquisition protocols, which may have resulted in adverse effects (37). Third, because of the limited experimental conditions and lack of enough funding, the significant correlations between imaging features and gene data could not be validated in molecular biology experiment and clinical trials. However, to our knowledge, this is the first study to predict OS for patients with GC by radiogenomics, which might provide valuable references in this field.

## Conclusion

In conclusion, in this study, we presented three imaging biomarkers with significant prognostic value and a nomogram prognostic model for patients with GC. The imaging biomarkers correlated with RNA expression profiles can provide possible biological interpretations and may facilitate a better understanding of the molecular characterization of GC. The proposed nomogram showed better prognostic performance than the TNM staging system. Researchers, clinicians, and patients can easily predict the survival probability using this nomogram.

## Data Availability Statement

The data that support the findings of this study are available from the corresponding author, TC, upon reasonable request.

## Author Contributions

Conceptualization: TC, HL, YyW, YqL, and WtK; Formal analysis: HL, YyW and LC; Funding acquisition: TC and WtK; Methodology, TC, HL, YyW, JyY, and ZC; Software: HL, YyW, DyL, YyZ, and YL; Supervision: TC, HL, JgY, and WtK; Writing—original draft: HL, YyW, WtK, and TC; Writing—review and editing: HL, TC, and WtK. All authors contributed to the article and approved the submitted version.

## Funding

This study was supported by Guangdong Provincial Key Laboratory of Precision Medicine for Gastrointestinal Cancer (2020B121201004), the Guangdong Natural Science Foundation General Project (2019A1515011520), Guangdong Natural Science Foundation Outstanding Youth Project (2021B1515020055), and Guangdong Provincial Major Talents Project (2019JC05Y361).

## Conflict of Interest

The authors declare that the research was conducted in the absence of any commercial or financial relationships that could be construed as a potential conflict of interest.

## Publisher’s Note

All claims expressed in this article are solely those of the authors and do not necessarily represent those of their affiliated organizations, or those of the publisher, the editors and the reviewers. Any product that may be evaluated in this article, or claim that may be made by its manufacturer, is not guaranteed or endorsed by the publisher.
