# Supplementary material for: Contrast-Enhanced Computed Tomography–Based Radiogenomics Analysis for Predicting Prognosis in Gastric Cancer
Source: Front Oncol. 2022 Jun 22;12:882786. doi: 10.3389/fonc.2022.882786 (PMC9257248; doi:10.3389/fonc.2022.882786)
Supplement: Supplementary file 1 [file DataSheet_1.docx]

**The expatiation of the four matrices**

The high-order neighbourhood grey-tone difference matrix (NGTDM) method computes the intensity differences between a voxel and its neighbours in 2D and can be adapted to 3D for 26 neighbours. It is a column matrix formed as follows.

Let$f(k,l)$be the gray tone of any pixel at$(k,l)$having gray tone value$i$. Then we find the average gray-tone over a neighborhood centered at, but excluding$(k,l)$

$$\bar{A}_{i}=\bar{A}(k,l)=\frac{1}{W-1}\left[\sum_{m=-dn=-d}^{d} f(k+m,l+n)\right]$$

where d specifies the neighborhood size and $W=(2d+1)^{2}$

Then the ith entry in the NGTDM is

$$s(i)=\sum\left|i-\bar{A}_{i}\right|$$

where $\left\{N_{i}\right\}$ is the set of all pixels having gray tone$i$(except in the peripheral regions of width$d$).

The Grey Level Size Zone Matrix (GLSZM) provides information on the size of homogeneous zones for each grey-level in 3 dimensions. It is also named grey-level zone length matrix (GLZLM)

Element $(i,j)$ of GLSZM corresponds to the number of homogeneous zones of $j$ voxels with the intensity ii in an image and is called GLSZM$(i,j)$ thereafter.

The grey-level run length matrix (GLRLM) gives the size of homogeneous runs for each grey level. This matrix is computed for the 13 different directions in 3D (4 in 2D) and for each of the 11 texture indices derived from this matrix, the 3D value is the average over the 13 directions in 3D (4 in 2D). The element$(i,j)$of GLRLM corresponds to the number of homogeneous runs of $j$ voxels with intensity ii in an image and is called GLRLM$(i,j)$ thereafter.

The grey level co-occurrence matrix (GLCM) takes into account the arrangements of pairs of voxels to calculate textural indices. The GLCM is calculated from 13 different directions in 3D with a $\delta-\text{voxel}$distance$(\text{‖}\vec{d}\text{‖})$relationship between neighboured voxels. The index value is the average of the index over the 13 directions in space (X, Y, Z). Six textural indices can be computed from this matrix. An entry $(i,j)$ of GLCM for one direction is equal to:

$$GLCM_{\Delta x,\Delta y}(i,j)=\frac{1}{\text{ Pairs }_{ROI}}\sum_{p=1}^{N-\Delta xM-\Delta y} \left\{\begin{matrix} 1\text{ if }(I(p,q)=i,I(p+\Delta x,q+\Delta y)=j) \\ \text{ and }I(p,q),I(p+\Delta x,q+\Delta y)\in ROI \\ 0\text{ otherwise } \end{matrix}\right.$$

where$I(p,q)$corresponds to voxel$(p,q)$in an image of size $N*M$. The vector$\vec{d}=(\Delta x,\Delta y)$covers the 4 directions (D1, D2, D3, D4) in 2D space or 13 directions.

**The calculation of the radiomics score (RIS)**

The RIS was calculated with the following equation:

RIS score=$\sum_{1}^{\text{n}} \text{β}$_i_ × M_i_

In the formula, n refers to the total number of radiomics features linked to overall survival, **β**_i_ represents the regression coefficient of feature i. M_i_ is the score of each texture feature.

**The expatiation of imaging features and radiogenomic correlation**

The texture feature NGTDM.Complexity was positively correlated with overall survival because the hazard ratio was less than one (HR=0.87). It has been reported that texture features extracted from the neighborhood gray-tone difference matrix (NGTDM) can significantly predict survival in patients with non-small cell lung cancer undergoing concurrent chemoradiation therapy. Complexity refers to the visual information content of a texture. A texture is considered complex if the information content is high. Again, a texture with a large number of sharp edges and/or lines may be considered complex. The “complex shape” has been identified as an accurately predicted semantic feature of non-small cell lung cancer. In addition, NGTDM.Complexity was correlated with the gene module MEgreen. The gene module MEgreen was enriched in olfactory receptor activity and type I interferon receptor binding. The expression levels of lncRNA olfactory receptors are significantly associated with lymphatic metastasis, the depth of cancer invasion, and distal metastasis in gastric cancer tissues. Type I interferons (IFNs) play a vital role in restricting proliferation, cancer surveillance and regulating the adaptive immune response. Moreover, the intratumoral expression levels of type I IFNs or IFN-stimulated genes correlate with favorable disease outcomes in several cohorts of cancer patients. Type I IFNs exert their effect via the type I IFN receptor, whose expression level influences the response to type-I interferons. In conclusion, the imaging feature NGTDM.Complexity, which refers to olfactory receptor activity and type I interferon receptor binding, was suggested to be the biomarker significantly correlated with OS.

The texture feature GLRLM.LRLGE was negatively correlated with overall survival because the hazard ratio was much greater than one (HR=25451.93). Long-run low gray-level emphasis (LRLGE) reflects the distribution of long homogeneous runs with low gray levels. Recent studies have shown that LRLGE can predict the tumor response to neoadjuvant chemoradiotherapy in esophageal cancer patients. LRLGE is higher for complete responders and lower for incomplete responders. Similar to the imaging feature NGTDM.Complexity, GLRLM.LRLGE was also correlated with the gene module MEgreen, which was enriched in olfactory receptor activity and type I interferon receptor binding. Thus, the texture feature GLRLM.LRLGE is also a biomarker of olfactory receptor activity and type I interferon receptor binding.

The texture feature GLSZM.ZP had significant negative prognostic value for OS because its hazard ratio was much greater than one (HR=3.497e+20). The gray-level size zone matrix (GLSZM) provides information on the size of homogeneous zones for each gray level in 3 dimensions. The zone percentage (ZP) reflects the homogeneity and distribution of the zone of an image. It is largest when the size of the zone is 1 for all gray levels. The ZP for local or regional heterogeneity characterization can provide both a high differentiation power in terms of patient response prediction and is robust with respect to the delineation method. A recent study found that the zone percentage of the gray-level size zone matrix (GLSZM.ZP) was able to predict nodal metastases in endometrial cancer, and tumors with nodal metastases are generally characterized by a lower GLSZM ZP value. In addition, GLSZM.ZP was associated with the gene module MEdarkturquoise, which is enriched in protein kinase activator activity. Protein kinase is an important group of drug targets, and protein kinase inhibitors are widely utilized in the clinic. A number of diseases, including cancer, diabetes, and inflammation, are linked to perturbations in protein kinase-mediated cell signaling pathways. Several kinds of protein kinases were strongly associated with gastric cancer. The mitogen-activated protein kinase (MAPK) played in the invasion and metastasis of gastric cancer. 3-phosphoinositide dependent protein kinase-1 (PDK-1) promoted cell viability, migration, invasion, and epithelial-mesenchymal transition in GC. And protein kinase Cι (PKCι) is correlated with tumor progression and angiogenesis and it is identified to be a new prognostic factor for gastric cancer. Thus, the texture feature “GLSZM.ZSV” was the prognostic biomarker correlated with protein kinase activator activity.

Not limited to the three biomarkers, we also identified other significant correlations between imaging features and gene modules. Though the prognostic ability of the other features was not observed, these features may provide useful information for us to further understand the biological interpretations of imaging traits.

For example, the first-order statistic volume.eccentricity was positively correlated with the gene module MEroyalblue (P=0.0163). The feature eccentricity was reported to have the best performance in the classification between benign and malignant ovarian masses and the prognosis of patients. The gene module MEroyalblue was enriched in microRNAs that guide posttranscriptional gene silencing in cancer. MicroRNA (miRNA) is involved in biological processes including cell proliferation, metastasis, differentiation, development and apoptosis. And in gastric cancer, MiRNA plays pivotal role in proliferation, apoptosis, cell invasion and metastasis. Additionally, MiRNA has been acknowledged to be the potential biomarkers and therapeutic targets for Gastric Cancer. Plenty of studies demonstrated an improved sensitivity and specificity than conventional tumor markers (CEA, CA12-5, CA19-9 and CA72-4). In summary, the first-order statistic volume.eccentricity was correlated with the expression of miRNAs and might provide new insights into the biology of various forms of cancer.

The texture feature GLCM.Entropy was negatively correlated with the gene module MEwhite (P=0.0353). Entropy represents the randomness of gray-level voxel pairs. A recent study of the radiomics of locally advanced cervical cancer identified GLCM.Entropy in ADC maps from DWI MRI as an independent prognostic factor that had significantly higher prognostic power than other clinical parameters. And in gastric cancer, Entropy was positively correlated with the receptor of vascular endothelial growth factor (VEGFR2) expression level which is principally responsible for mediating the mitogenic-, angiogenic- and permeability-enhancing effects of VEGF and potentially plays a role in stimulating tumour growth and metastasis. The gene module MEwhite was enriched in nicotinamide adenine dinucleotide hydride (NADH) dehydrogenase activity. Mitochondrial NADH dehydrogenase subunit 3 (MTND3) polymorphisms was proved to have an association with gastric cancer susceptibility and altered expression of NADH dehydrogenase 4 transcripts was associated With gastric tumorigenesis and tumor dedifferentiation. In conclusion, the texture feature GLCM.Entropy was correlated with NADH dehydrogenase activity and could provide positive prognostic information for clinicians. The mechanisms deserve further investigation.

**Immune correlation of imaging biomarkers/RIS**

Based on TCGA transcriptional profiles from GC patients with CECT imaging data, we calculated the ratios of 22 types of tumor-infiltrating immune cells and the result showed that RIS_high subgroup was associated with higher CD8+ T cell、CD4+ memory T cells infiltration in tumor microenvironment (P<0.05) (Figure S5A、B). It indicated that the activation of immune components in the tumor microenvironment may contribute to the worse prognosis in high-risk patients. Figure S5C illustrated some significant co-expression patterns about immune cells. RIS_high subgroup showed a higher immunophenoscore z-score (immunophenoscore was used to characterize the intratumoral immune landscapes and the cancer antigenomes) than those in RIS_low subgroup, suggesting that patients with lower RIS may be more sensitive to immune checkpoint inhibitor treatment (Figure S5D). Morever, we also calculated the correlation between tumor-infiltrating cells and the selected imaging features mentioned above. As shown in the correlation heatmap，CD8+ T cell was negatively correlated with NGTDM.Complexity and GLRLM.LRLGE, and an apparent negative correlation can be seen between CD4+ memory activated T cells and NGTDM.Complexity (Figure S5E). The heterogeneity of immune infiltration in prognostic imaging features may provide prognostic indicators and may provide deep interpretation for the imaging biomarkers.

**Supplementary Table S1.**Quantitative imaging features

| Type | feature | description |
| --- | --- | --- |
| First-order statistic | variance | the degree differ from mean. |
|  | kurtosis | the histogram sharpness. |
|  | skewness | the histogram asymmetry. |
|  | entropy | the histogram randomness. |
| Texture(GLCM) |  | the Gray-Level Co-occurence Matrix of ROI. |
|  | Energy | the uniformity of grey-level voxel pairs. |
|  | Contrast | The distribution of the values of the metric matrix and the local variations of the image reflect the sharpness of the image and the depth of the texture. |
|  | Entropy | the randomness of grey-level voxel pairs. |
|  | Homogeneity | the homogeneity of grey-level voxel pairs. |
|  | Correlation | the linear dependency of grey-levels in GLCM. |
|  | SumAverage | measures the relationship between occurrences of pairs with lower intensity values and occurrences of pairs with higher intensity values. |
|  | Variance | the local variations in the GLCM. |
|  | Dissimilarity | the variation of grey-level voxel pairs. |
|  | AutoCorrelation | a measure of the magnitude of the fineness and coarseness of texture. |
| Texture(GLRLM) |  | the Gray-Level Run-Length Matrix of ROI. |
|  | SRE | the distribution of the short homogeneous runs in an image. |
|  | LRE | the distribution of the long homogeneous runs in an image. |
|  | GLN | the non-uniformity of the grey-levels. |
|  | RLN | the non-uniformity of the length of the homogeneous runs. |
|  | RP | Run Percentage, measures the homogeneity of the homogeneous runs. |
|  | LGRE | the distribution of the low grey-level runs. |
|  | HGRE | the distribution of the high grey-level runs. |
|  | SRLGE | the distribution of the short homogeneous runs with low grey-levels. |
|  | SRHGE | the distribution of the short homogeneous runs with high grey-levels. |
|  | LRLGE | the distribution of the long homogeneous runs with low grey-levels. |
|  | LRHGE | the distribution of the long homogeneous runs with high grey-levels. |
|  | GLV | measures the variance in gray level intensity for the runs. |
|  | RLV | measures the variance in runs for the run lengths. |
| Texture(GLSZM) |  | the Gray-Level Size Zone Matrix of ROI. |
|  | SZE | the distribution of the short homogeneous zones in an image. |
|  | LZE | the distribution of the long homogeneous zones in an image. |
|  | GLN | the non-uniformity of the grey-levels. |
|  | ZSN | the non-uniformity of the length of the homogeneous zones. |
|  | ZP | Zone Percentage measures the homogeneity of the homogeneous zones. |
|  | LGZE | the distribution of the low grey-level zones. |
|  | HGZE | the distribution of the high grey-level zones. |
|  | SZLGE | the distribution of the short homogeneous zones with low grey-levels. |
|  | SZHGE | the distribution of the short homogeneous zones with high grey-levels. |
|  | LZLGE | the distribution of the long homogeneous zones with low grey-levels. |
|  | LZHGE | the distribution of the long homogeneous zones with high grey-levels. |
|  | GLV | measures the variance in gray level intensities for the zones. |
|  | ZSV | measures the variance in zone size volumes for the zones. |
| Texture(NGTDM) |  | the Neighborhood Gray-Tone Difference Matrix of ROI. |
|  | Coarseness | the level of spatial rate of change in intensity. |
|  | Contrast | the intensity difference between neighbouring regions. |
|  | Busyness | the spatial frequency of changes in intensity. |
|  | Complexity | refers to the visual information content of a texture. A texture is considered complex if the information content is high. |
|  | Strength | measures the primitives in an image. Its value is high when the primitives are easily defined and visible, an image with slow change in intensity but more large coarse differences in gray level intensities. |

**Supplementary Table S2**.Annotations of prognostic gene modules in GO enrichment analysis and KEGG pathways.

| Gene Module | GO term (Biological process)(adjust p<0.05) | KEGG pathway(adjust P<0.05) |
| --- | --- | --- |
| Medarkturquoise | receptor agonist activity |  |
|  | protein kinase activator activity |  |
|  | kinase activator activity |  |
| Megreen | olfactory receptor activity | Olfactory transduction |
|  | odorant binding |  |
|  | mRNA binding involved in posttranscriptional gene silencing |  |
|  | type I interferon receptor binding |  |
|  | mRNA binding |  |
| Meroyalblue | mRNA binding involved in posttranscriptional gene silencing | MicroRNAs in cancer |
|  | microtubule binding |  |
|  | tubulin binding |  |
|  | mRNA binding |  |
| Mewhite | NADH dehydrogenase (ubiquinone) activity | Ribosome biogenesis in eukaryotes |
|  | NADH dehydrogenase (quinone) activity | Ribosome |
|  | NADH dehydrogenase activity |  |
|  | oxidoreductase activity, acting on NAD(P)H, quinone or similar compound as acceptor |  |
|  | oxidoreductase activity, acting on NAD(P)H |  |

**Supplementary Table S3** Multiple Cox regression coeﬃcients and hazard ratio for survival-related imaging features in TCGA database

|  | **Hazard ratio** | **HR0.95L** | **HR0.95H** | **P value** |
| --- | --- | --- | --- | --- |
| NGTDM.Complexity | 0.8372 | 0.7551 | 0.9283 | 0.001 |
| GLRLM.LRLGE | 84.07 | 0.006826 | 1035000 | 0.36 |
| GLSZM.ZP | 5.030e+25 | 3.094e+08 | 8.180e+42 | 0.003 |

**Supplementary Table S4.** Cox regression coeﬃcients and nomogram score from the training cohort

|  | **Cox regression coeﬃcients** | **Nomogram score** |
| --- | --- | --- |
| **RIS_score^a^** | 0.77 | 22.22*RIS + 55.56 |
| **Age^a^** | 0.03 | 0.8932*Age-17.8643 |
| **Lymphv^a^** | 1.40 | 40.6*Lymphv |
| **fT** |  |  |
| T1 | 0 | 0 |
| T2 | 1.53 | 44 |
| T3 | 1.89 | 55 |
| T4 | 2.30 | 67 |
| **fM** |  |  |
| M0 | 0 | 0 |
| M1 | 1.09 | 31 |

^a^ Continuous variable

**Supplementary Figure S1.**Tumor delineation of four samples

**
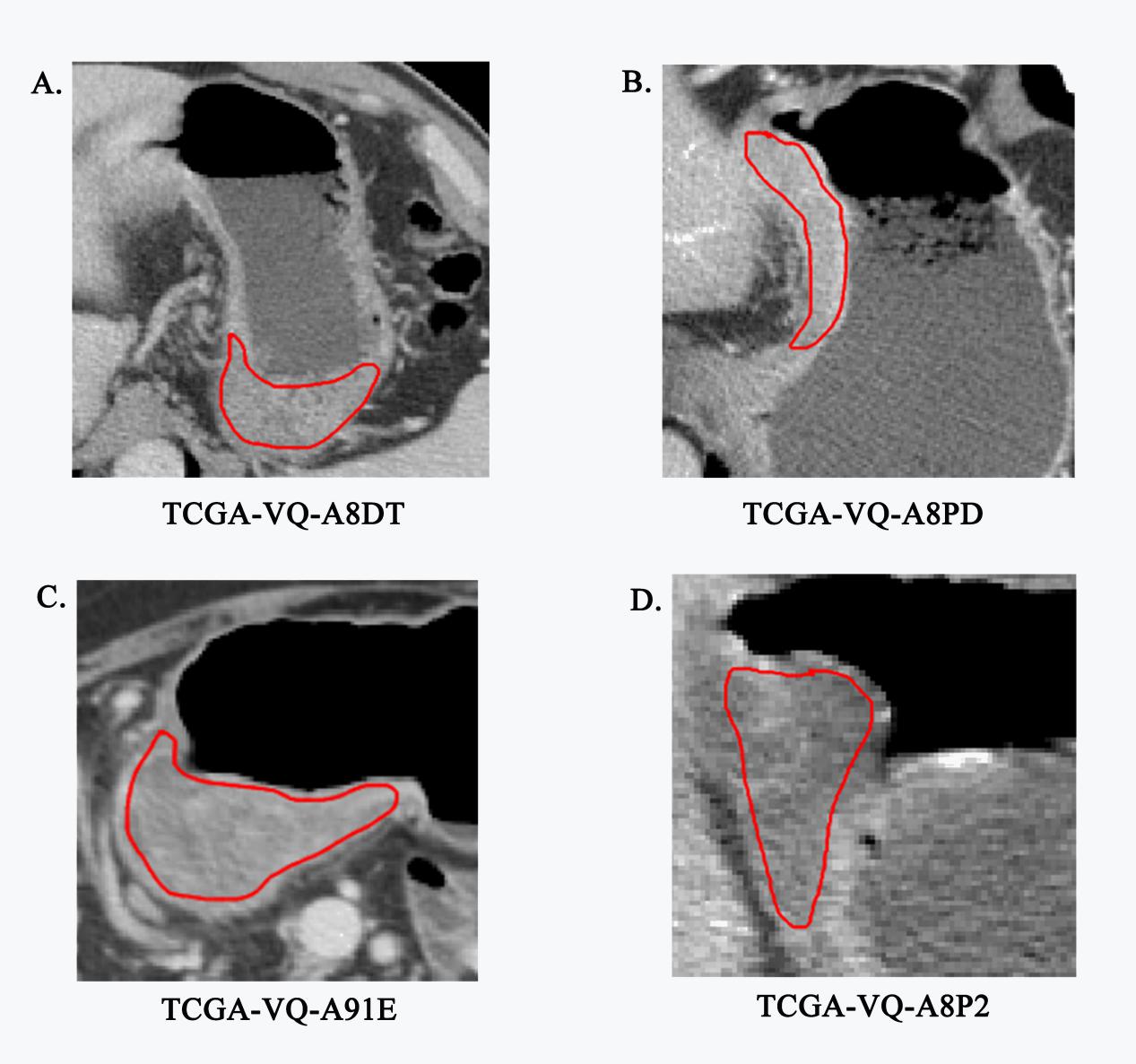
**

**Supplementary Figure S2.**The correlation matrix of 28 robust imaging features


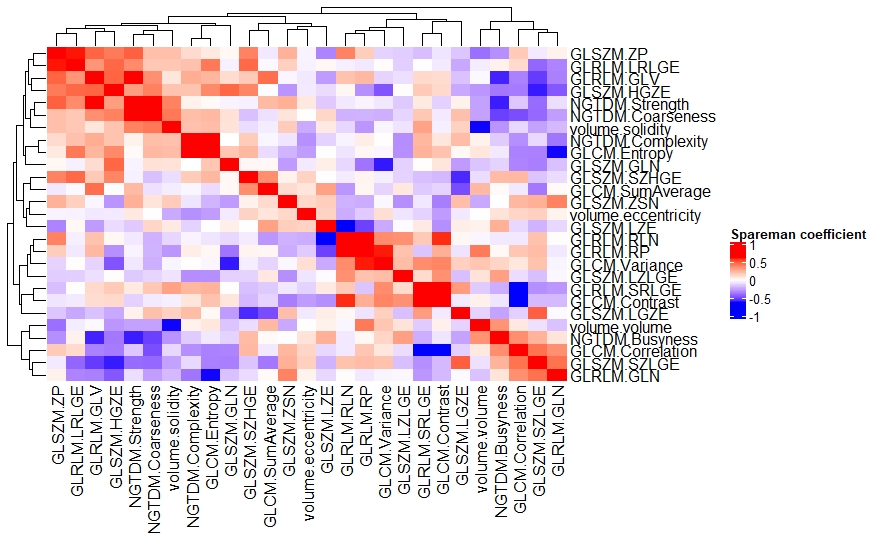


**Supplementary Figure S3A.** Consensus gene dendrogram of 58,428 genes for 407 GC patients and module colors.

**
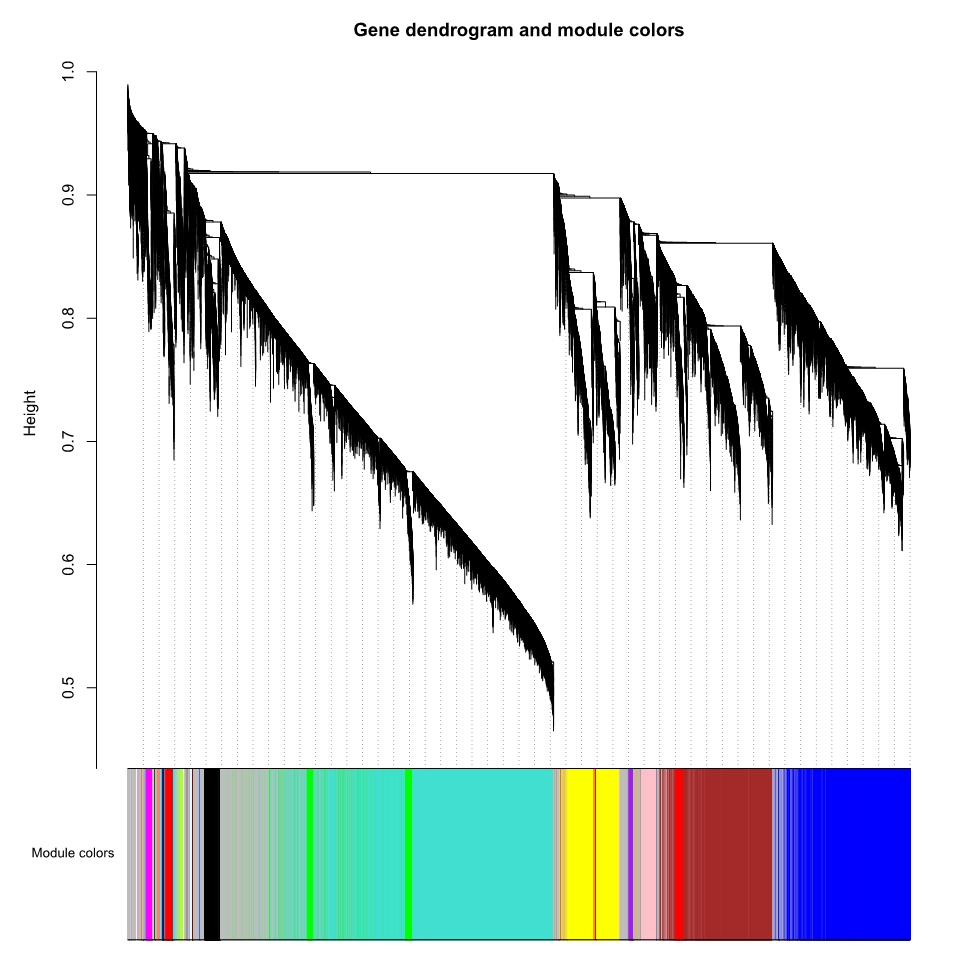
**

**Supplementary Figure S3B/C.** Analysis of network topology for various soft-thresholding powers


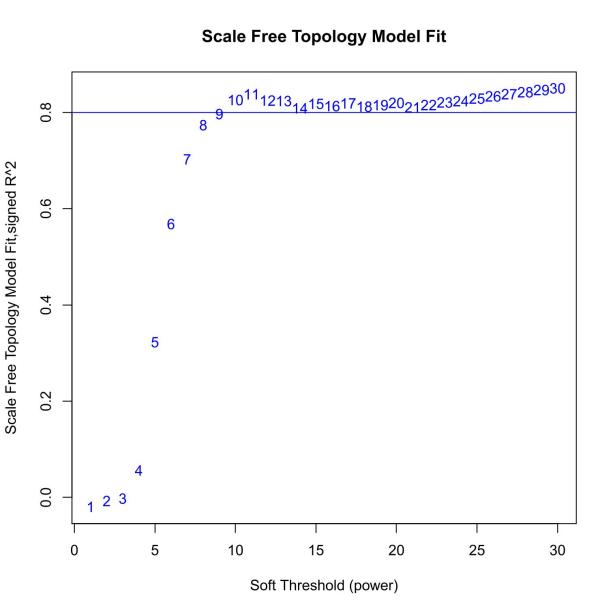

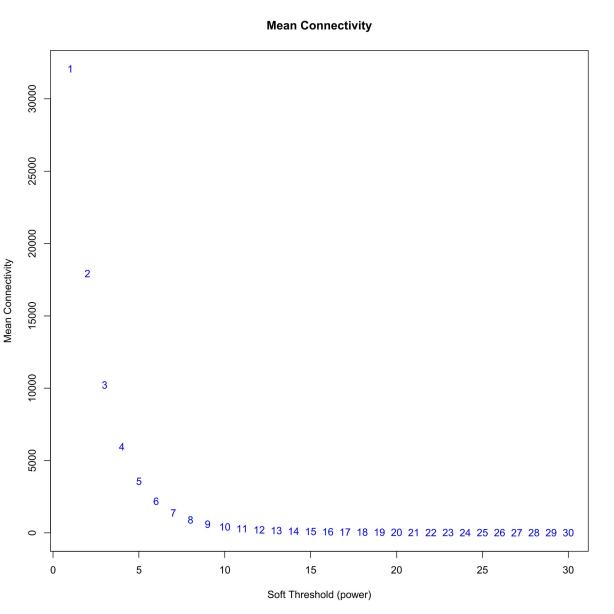


1. (C)

(B)The x-axis reflects the soft-thresholding power. The y-axis reflects the scale-free topology model fit index. (C) The x-axis reflects the soft-thresholding power. The y-axis reflects the mean connectivity (degree).

**Supplementary Figure S4.** Validation of the prediction model.


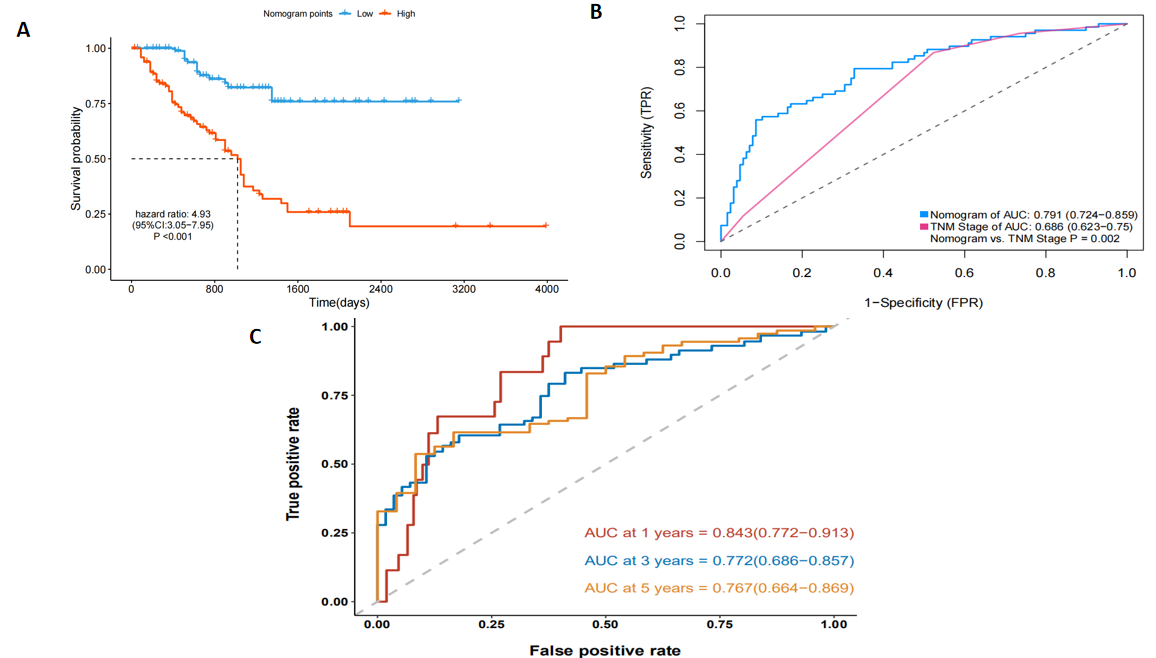


1. Kaplan–Meier curves for patients with high- and low nomogram score in the validation cohort. (B) The ROC curve to evaluate the nomogram model and TNM stage in the validation cohort. (C) ROC of the nomogram model in the validation cohort.The area under the ROC curve was 0.815, 0.780 and 0.790 for the nomogram score at 1, 3 and 5 years, respectively.

**Supplementary Figure S5.**Tumor-infiltrating cells profiles in GC patients with CECT imaging data

**
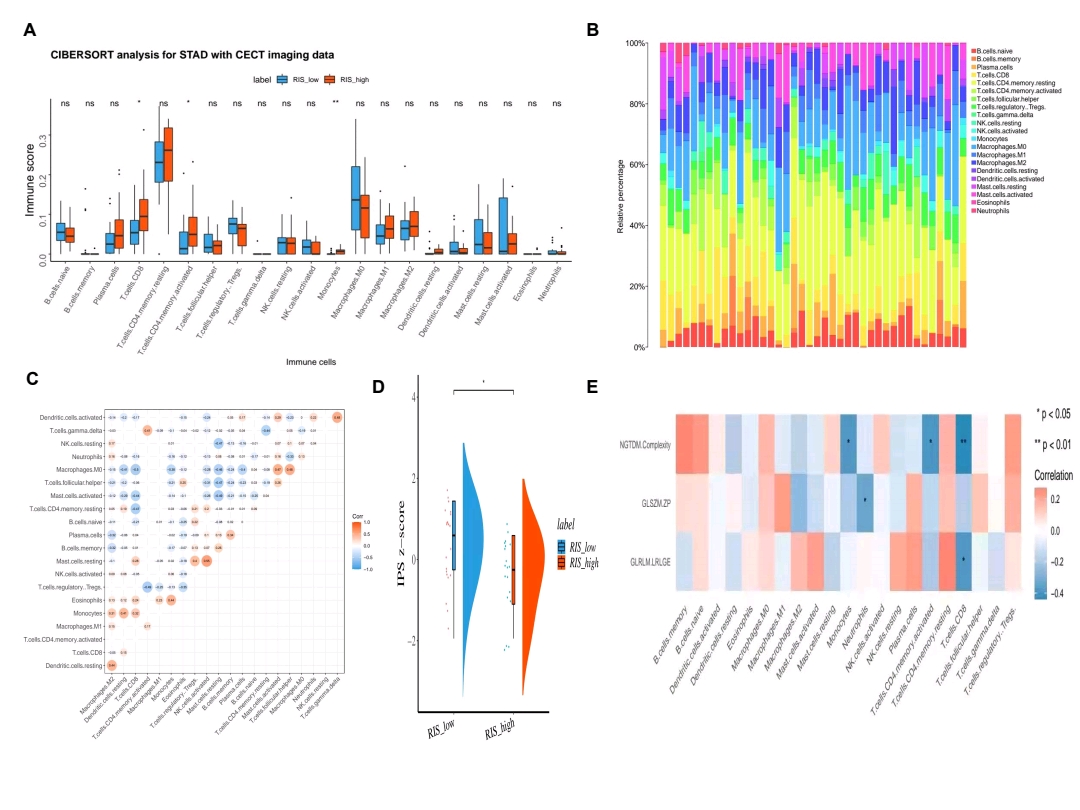
**

1. Comparison of RIS subtypes based on 21 immune cell types. (B) Distribution of 21 immune cells in GC patients with CECT imaging data. (C) Heatmap showing the correlation between 21 immune cells and the size of each tiny circle indicating the p value of correlation between two kinds of immune cells (D) Boxplot showed the aggregated IPS z-score of two radiomics subgroups (E) The correlation analysis between tumor-infiltrating cells and selected imaging features. The co-expression heatmap visualized the results.
