# Supplementary figures and images for: Contrast-Enhanced Computed Tomography–Based Radiogenomics Analysis for Predicting Prognosis in Gastric Cancer
Source: Front Oncol. 2022 Jun 22;12:882786. doi: 10.3389/fonc.2022.882786 (PMC9257248; doi:10.3389/fonc.2022.882786)

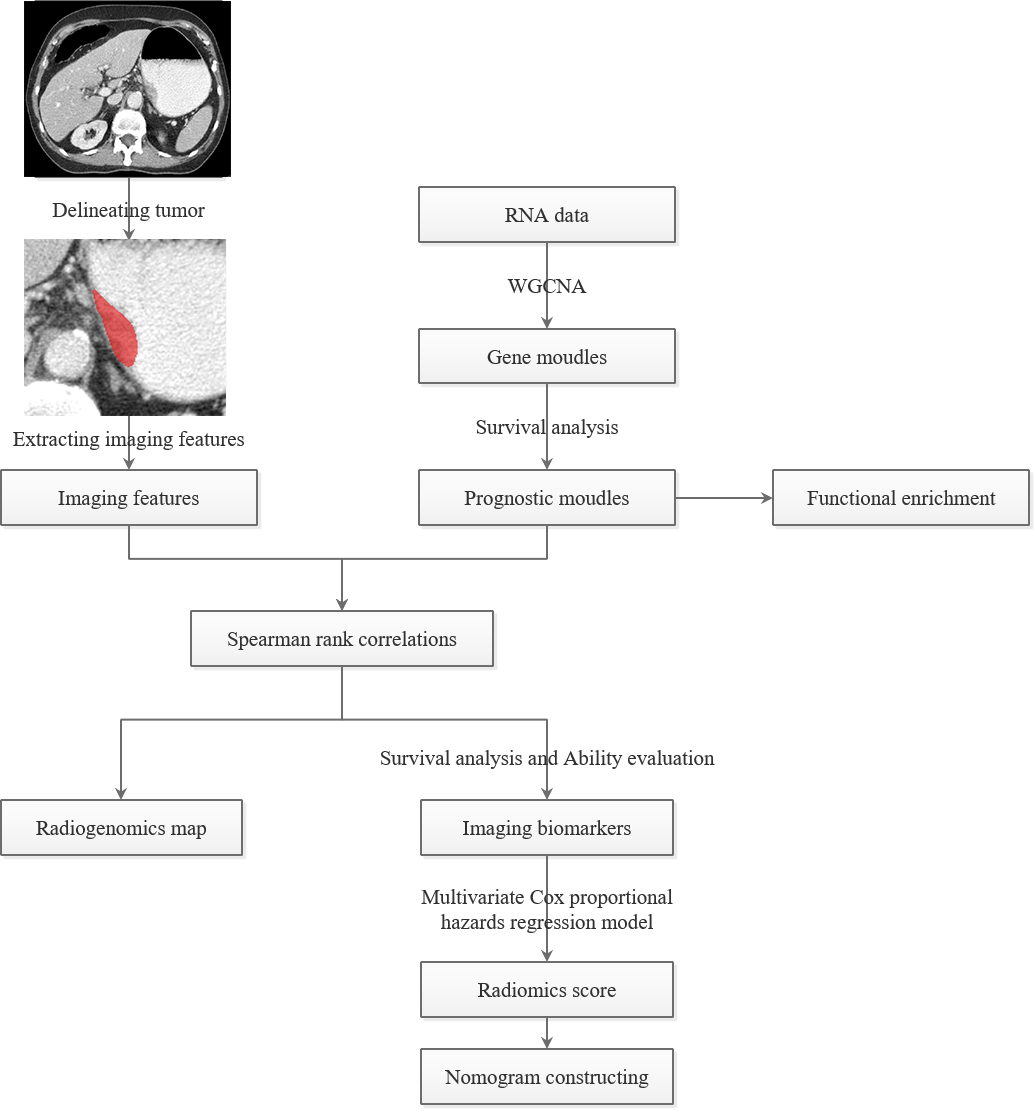

Supplement: Supplementary file 2 [file Image_1.jpeg]
